# The Potential Application of Green-Synthesized Metal Nanoparticles in Dentistry: A Comprehensive Review

**DOI:** 10.1155/2022/2311910

**Published:** 2022-03-03

**Authors:** Mohsen Yazdanian, Pouya Rostamzadeh, Mahdi Rahbar, Mostafa Alam, Kamyar Abbasi, Elahe Tahmasebi, Hamid Tebyaniyan, Reza Ranjbar, Alexander Seifalian, Alireza Yazdanian

**Affiliations:** ^1^Research Center for Prevention of Oral and Dental Diseases, Baqiyatallah University of Medical Sciences, Tehran, Iran; ^2^School of Dentistry, Baqiyatallah University of Medical Sciences, Tehran, Iran; ^3^Dental Students' Scientific Research Center (DSSRC), Tehran University of Medical Sciences, Tehran, Iran; ^4^Department of Restorative Dentistry, School of Dentistry, Ardabil University of Medical Sciences, Ardabil, Iran; ^5^Department of Oral and Maxillofacial Surgery, School of Dentistry, Shahid Beheshti University of Medical Sciences, Tehran, Iran; ^6^Department of Prosthodontics, School of Dentistry, Shahid Beheshti University of Medical Sciences, Tehran, Iran; ^7^Science and Research Branch, Islamic Azad University, Tehran, Iran; ^8^Nanotechnology and Regenerative Medicine Commercialization Centre (NanoRegMed Ltd), The London Bioscience Innovation Centre, London, UK; ^9^Department of Veterinary, Science and Research Branch, Islamic Azad University, Tehran, Iran

## Abstract

Orodental problems have long been managed using herbal medicine. The development of nanoparticle formulations with herbal medicine has now become a breakthrough in dentistry because the synthesis of biogenic metal nanoparticles (MNPs) using plant extracts can address the drawbacks of herbal treatments. Green production of MNPs such as Ag, Au, and Fe nanoparticles enhanced by plant extracts has been proven to be beneficial in managing numerous orodental disorders, even outperforming traditional materials. Nanostructures are utilized in dental advances and diagnostics. Oral disease prevention medicines, prostheses, and tooth implantation all employ nanoparticles. Nanomaterials can also deliver oral fluid or pharmaceuticals, treating oral cancers and providing a high level of oral healthcare. These are also found in toothpaste, mouthwash, and other dental care products. However, there is a lack of understanding about the safety of nanomaterials, necessitating additional study. Many problems, including medication resistance, might be addressed using nanoparticles produced by green synthesis. This study reviews the green synthesis of MNPs applied in dentistry in recent studies (2010–2021).

## 1. Introduction

**Figure 1:**
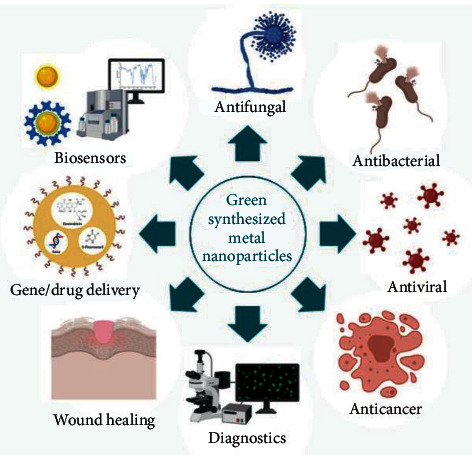
Green-synthesized metal nanoparticles' medical applications [10].

Nanotechnology is a field of science that deals with nanometer-sized objects, which are referred to as nanoparticles (NPs). Nanomaterials are small solid particles having a dimension of 1–100 nanometers. Nanomaterials show promise in antibacterial therapy because of their improved and distinct physicochemical properties, including very small dimensions, huge surface area compared to their mass, and higher reactivity [1–5]. By adding many functional groups to the nanoparticle, the quality of products can be improved. Therefore, nanoproducts are widely used in different industrial, medical, and dentistry sectors. Nanobiotechnology is a unique method that has inspired the development of a variety of nanobiomaterials with applications in biology and medicine [6]. Despite their potential antibacterial properties, most methods for synthesizing these nanoparticles are costly and may have negative consequences for the environment, biological systems, and human health due to the usage of toxic and dangerous substances. As a result, “green” nanoparticle synthetization technologies have been created. Because no hazardous compounds are utilized, this alternative uses biological systems such as yeast, fungus, bacteria, and plant extracts, making it a safer and more environmentally friendly alternative to chemical approaches. Plant extracts are widely used for a variety of reasons, including their enormous and accessible reserves, global distribution, safe handling, availability of a diverse range of metabolites with high reducing potentials, and low waste and energy costs [7–9]. The broad application of medical nanosystems in different branches of dentistry, including prognosis, prevention, tissue regeneration, repair, and care, has been documented in numerous studies (Figure 1). For quality oral care, advancements in oral medicine nanosystems for individual prophylaxis are critical. Due to their broad-spectrum antibacterial capabilities, metal nanoparticles (MNPs) have been used in various dental applications. To achieve a greater antibacterial impact, tinier MNPs might release more of their ions. Many studies on the antibacterial activities of NPs have found that NPs have greater antibacterial activity in bacteria that are resistant to antibiotics. As a result, the use of nanoparticles in dentistry could be very beneficial [11–13]. Oral cosmetics with nanomaterials are used in toothpaste and other products to promote oral health. These procedures are applicable to nanoparticles and nanoparticle-based materials, with a focus on plaque management in periodontology and cariology. NPs have also been used in a variety of cosmetic products to help with enamel remineralization and dental hypersensitivity [14]. More than 75 bacterial and fungal strains have been linked to oral disorders. The oral microbiota had been altered by the accessible chemical reagents, resulting in diarrhea, vomiting, and tooth discoloration. Traditional medications can have a role in antibiotic treatment in general, but antibiotic resistance and undesirable side effects such as hypersensitivity, immunological suppression, and allergic reactions are growing concerns. As a result, scientists are attempting to create novel goods using natural materials. Plant-based biomolecules can inhibit the growth of oral infections, reduce tooth plaque, and reduce the symptoms of oral illnesses [15]. Biofilms are characterized as microbial communities that may house many bacterial and fungal species and are associated with nearly every surface on the planet, including human hard and soft tissues, and are embedded in extracellular polymeric substances. The accumulation of acidogenic biofilms on tooth surfaces, in particular, causes the enamel to dissolve, a process known as demineralization, which, if left untreated for long periods of time, can cause the development of caries. Controlling oral biofilm production is a difficult challenge, but nanotherapeutics has been employed successfully in recent years by adding nanoparticles into a variety of dental materials [7]. In this study, the dental application of green synthesis (GS) in the production of MNPs has been reviewed in recent studies (2010–2021).

## 2. Metal Nanoparticles

MNPs are the most widely used inorganic NPs and can be considered a viable solution to antibiotic resistance. Furthermore, they attack a variety of biomolecules, posing a threat to the formation of resistant strains [16]. Because of their physicochemical properties and uses in biotechnology, metal nanoparticles (MNPs) created using green methods have risen in popularity. Nowadays, green-synthesizing NPs from plant extracts have become a critical concentration of researchers due to the low toxicity of these NPs in the human body and minimal hazardous influence on the environment. The shape and size of plant-derived NPs are more stable, and they yield more than the other approaches. Furthermore, some of these MNPs have demonstrated antibacterial action, regularly validated in recent years. Plant extracts have been employed as a reducing (RA) and stabilizer agent (SA) of NPs, allowing us to minimize toxicity in both the environment and the human body without the need for chemical agents [17].

### 2.1. Metal Nanoparticles' Characterization

Nanoparticles have been studied using a variety of approaches to determine their size, crystal structure, elemental content, and a range of other physical features. Physical attributes can be examined using more than one technique in numerous instances. Different strengths and limits of each methodology make selecting the best method difficult, and a combinatorial characterization is frequently required. Size and shape are two of the essential criteria addressed in the characterization of NPs. We may also assess the surface chemistry and estimate the size distribution, degree of aggregation, surface charge, and surface area. Other features and applications of NPs may be influenced by their size, size distribution, and organic ligands on their surfaces [18–20]. There are microscopy-based techniques (e.g., confocal laser scanning microscopy (CLSM), scanning electron microscopy (SEM), transmission electron microscopy [21], and atomic force microscopy (AFM)) [22], which provide information on the nanomaterials' size, shape, and crystal structure. Other approaches, such as magnetic procedures, are tailored to certain families of materials. SQUID, VSM, FMR, and XMCD are examples of these approaches. Many more techniques give further information on the nanoparticle samples' structure, elemental content, optical characteristics, and other common and more particular physical qualities. X-ray, spectroscopy, and scattering techniques are examples of these techniques. Microstructure and dispersion (sizes and spatial distribution) of NPs must be described as a function of different process parameters to optimize the material qualities of MNPs [23]. UV/visible spectroscopy is a method for determining how much light is absorbed and dispersed by a substance. UV/Vis spectroscopy is a valuable method for identifying, characterizing, and investigating gold and silver plasmonic nanoparticles because their optical properties are sensitive to size, shape, concentration, agglomeration state, and refractive index near the nanoparticle surface. Transmission electron microscopy [21] is a high-magnification imaging technique that records the transmission of an electron beam through a sample. The preferred way for directly measuring the particle size, grain size, size distribution, and morphology of nanoparticles is to use TEM imaging. Sizing precision is usually within 3% of the actual value. DLS (dynamic light scattering) is a valuable technology for determining the properties of nanoparticles and other colloidal solutions. Because it offers information on the aggregation state of nanoparticle solutions, the hydrodynamic diameter is a valuable complement to other size studies such as TEM [24].

### 2.2. Metal Nanoparticle Antibacterial Mechanisms

Electrostatic interactions draw electropositive MNPs to the surface of electronegative bacterial cell walls. Apart from this, MNPs form a strong bond with membranes, resulting in the breakdown of cell walls and enhanced permeability. Furthermore, nanoparticles can transfer metal ions [25] into the cell from the extracellular area, disrupting physiological systems. MIs and NPs may produce reactive oxygen species (ROS) in the intracellular space. The oxidative stress causes glutathione to be oxidized, reducing bacteria's antioxidant defense system against ROS. As a result, MIs can interact with cell components (membranes, proteins, and DNA), disrupting cell functions [15]. MIs can create strong coordination bonds with the nitrogen, oxygen, and sulfur atoms found in organic compounds and biomolecules. MNPs have a broad spectrum of activity because the connections between MIs and biological molecules are often not specific [26].

### 2.3. Disadvantages of the Application of Metal Nanoparticles

Although NPs have been shown to have numerous advantages, they also have certain drawbacks, such as high costs, simple inhalation of nanoparticles, which can lead to lung disease, and changes in homeostasis. Nanotoxicity is a novel discipline of toxicology that studies the side effects of NPs, which may have toxicological consequences. The nanoparticles' tiny size makes them highly reactive and causes many adverse molecular effects. Most plant extract nanoparticles are unprocessed, yet they are not the functional molecules of choice for plant extracts. For the low-cost production of nanoparticles, all functional groups of plant extracts are analyzed. Analyzing which molecule is employed as a RA or SA and identifying the biological nanoparticles in charge of therapeutic purposes are quite complex. Assessing the system's overall toxicity *in vivo* should be a top goal. The compensating dose for green-produced nanomaterials will be challenging to achieve with the reported dose. Green nanoparticles' long-term impacts on many clinicians will require more research in the future [27].

## 3. Approaches for NPs' Synthesis

### 3.1. Chemical Approach

**Figure 2:**
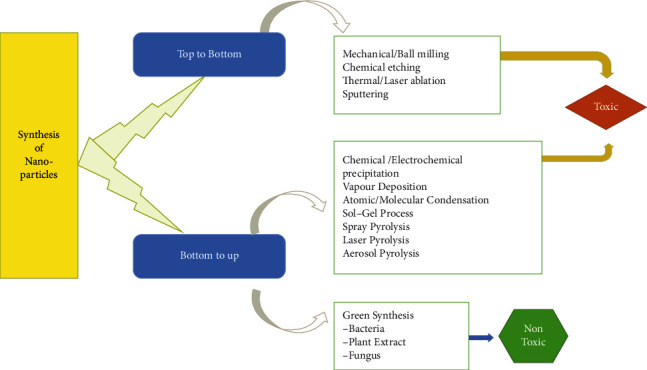
Various physical, chemical, and green approaches for the synthesis of NPs [29].

Metallic precursors [28], RA, and SA are the primary components of the chemical method (inorganic and organic). Elemental hydrogen, the polyol process, ascorbate, sodium citrate, NaBH_4_, Tollens' reagent, and ethylene glycol-block copolymers are all utilized as RAs (Figure 2) [30].

### 3.2. Physical Approach

The most common physical method for the production of nanoparticles is a “top-down” mechanism in which the size of the material decreased using techniques such as ultrasonication, microwave (MW) irradiation, and electrochemical methods (Figure 2). The most well-known physical mechanisms are laser removal and evaporation condensation. A carrier gas is created by vaporizing the material inside a pontoon focused on the heater. Various Au, Ag, and Cd NPs have been produced and published using this dissipation buildup approach [31].

### 3.3. Green Approach

**Figure 3:**
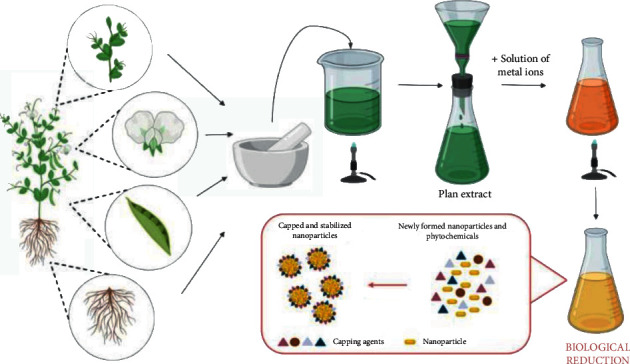
The typical process of plant-mediated green synthesis of metal nanoparticles [10].

Traditional techniques have long been utilized, but studies have shown that GS is the most successful method for creating NPs because it has fewer risks of failure, is less expensive, and is easier to characterize. GS particles are distinct from those created by physical and chemical methods. GS, a bottom-up mechanism for creating MNPs, is similar to the chemical approach, in which biological components such as a plant extract replace a costly chemical RA. Biological organisms have great potential for producing NPs. Green reduction of MPs to NPs is favorable to the environment and is sustainable, chemical-free, less expensive, and scalable. Furthermore, the GS of NPs leads to the recycling of valuable metal salts such as Au and Ag present in steams of waste. Greenly coordinated NPs are presently preferred over conventionally supplied NPs due to their superior qualities. Because of their insecurity and ambiguous composition, additional chemicals that are hazardous and poisonous to human health and the environment might enhance particle reactivity and toxicity and produce undesired adverse health impacts. Green synthesis approaches are appealing because they can lessen nanoparticles' toxicity. As a result, the usage of plant extracts is becoming increasingly popular (Figure 2) [32, 33]. The collection and purification of the plant component of interest is the initial step in a typical plant-mediated metal nanoparticle production. Then, the plant is dried and pulverized. Deionized distilled water is generally poured into the plant powder according to the required concentration for plant extract production. This solution is then heated before being filtered. A particular volume of the extract is combined with the right amount of metal salt solution. The combination is heated to the required temperature for the specified duration while being thoroughly mixed. A color shift of the solution is achieved when metal ions are reduced to metal nanoparticles which may then be checked by UV-visible spectra (Figure 3) [10]. The dentistry application of green synthesis of plant-mediated metal is summarized in Table 1.

#### 3.3.1. Benefits of Plant-Delivered Green Synthesis over Microorganism-Delivered Methods

The reaction rate is relatively high in plant extract-based synthesis methods. Depending on the type of plant and the amount of plant, this reaction takes a few minutes and several hours, but a considerable time (2 or many days) is necessary for microbe cultivation in the microorganism-based approaches. This shows that this is a strategy that takes time. In addition to these microbes, some are pretty hazardous and pose a hazard to human health. Still, most of these are safe and benign to generate nanoparticles, such as *Pseudomonas, Fusarium, and E. coli*. Many plants are nearly always available in nature, particularly evergreen ones. Metal nanoparticles are synthesized by plant extracts mostly at ambient temperature, whereas the reaction mixture and culture medium must be heated when microorganisms synthesize metallic nanoparticles. Plant extracts, rather than microbes, are better suitable for mass production [86]. The most critical metal nanoparticles and their plant-based green synthesis and application in dentistry will be discussed in the following sections.

## 4. Silver Nanoparticles

**Figure 4:**
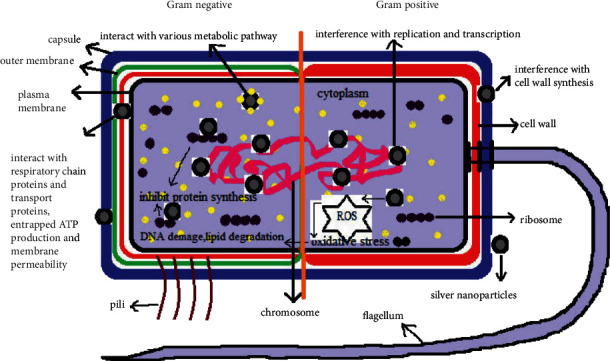
Antibacterial mechanism of silver nanoparticles [87].

**Figure 5:**
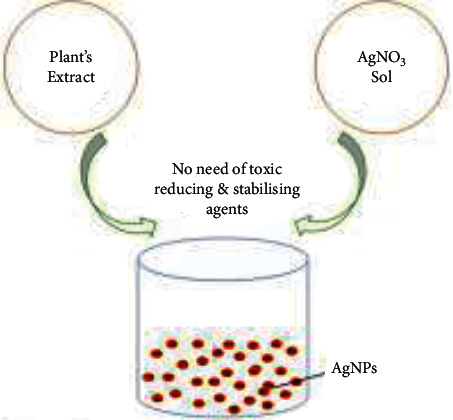
Green synthesis of AgNPs [29].

**Figure 6:**
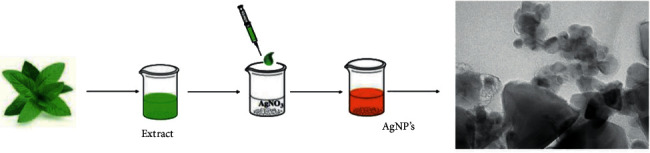
Schematic figure of green synthesis of AgNPs [88].

Silver nanoparticles (AgNPs) have attracted commercial interest due to various characteristics, including a changeable surface-area-to-volume ratio, helpful in various biological and technological applications. They are widely used in the electrical industry and serve as effective catalysts. Many papers reveal their biological activity in medical applications, such as anticancer, antioxidant, and antibacterial effects. Silver has been utilized historically from ancient times, and it has been proven that silver is harmless to human cells in low doses. Several action mechanisms have been suggested in antibacterial AgNP activities, for example, the potential of AgNPs to attach bacterial walls and to cause structural changes in the cell membrane, the ability to damage and porous the cell-based membrane as a result of free AgNP radicals, and the ability to release silver ions in the inner cell to destruct various functions in the cell (Figure 4). An antifungal mechanism of AgNPs was earlier postulated against *C. albicans* in that AgNPs had high potential for disturbing the cell membrane and stopping the G2/M cell cycle of *C. albicans* [40]. Previously, AgNPs mainly were made through a chemical procedure involving the reduction of silver nitrate (AgNO_3_) by a chemical reducing agent. Environmental resources, such as bacteria, plants, algae, and fungi, use the organic processes. The AgNPs' synthesis of microorganisms is easily scalable and naturally environmentally beneficial, although microorganism manufacturing is more expensive than plant extracts [40]. Plant extracts are used to make AgNPs because they include a lot of flavonoids, carbohydrates, sapogenins, and steroids which act as RA and biocapping chemicals that prevent nanoparticles from clumping together and allowing for greater size control (Figure 5). In general, obtaining AgNPs from plant extracts is a straightforward procedure. Plant fragments are gathered, sterile water cleaned, dried in the shade, and pulverized. The dried powder is boiled in deionized water to create the plant extract. The resulting infusion is filtered to remove any insoluble components. The solution containing 1 mM AgNO_3_ is then supplemented with a particular volume of the plant extract. The color change of the medium (typically to dark brown) and the ultraviolet-visible (UV-Vis) spectra can be used to confirm the AgNPs' synthesis reaction. Repeated centrifugation procedures at 12,000 rpm for 15 minutes will easily collect AgNPs (Figure 6) [29].

Pomegranate (*Punica granatum* L.) has long been utilized as a reducing agent for Ag^+^ ions. Pomegranate is known for its high phenolic content, including punicalagin, punicalin, ellagitannins, gallic acid, ellagic acid, and anthocyanins, which have anti-inflammatory qualities. Polyphenols, such as ellagic acid and gallic acid, are considered the elements responsible for the decrease of Ag^+^ ions and the stabilization of AgNPs [7]. Rice (*Oryza sativa* L.) is a Poaceae family cereal plant. The rice husk is hard to preserve the kernel inside the rice grain [39]. The leftover product contains the interior endosperm as well as the exterior rice bran (RB) and rice germ (RG) after RH is removed. Many sections of the rice grain contain a high concentration of antioxidant and reducing active compounds. Suwan et al. used the rice extract to make AgNPs. They demonstrated that RB, RH, and RG aqueous extracts may be employed as reducing agents in the manufacture of silver nanoparticles (AgNPs). Their antimicrobial studies revealed that AgNPs derived from green synthesis catalyzed by rice extracts exhibit potent antibacterial action against *S. mutans*, a serious oral infection that causes caries. RB is the most effective and suitable component of the rice grain for AgNP production out of the three sections [40]. Jain and Mehata used green chemistry to make AgNPs using *Ocimum sanctum* (tulsi) leaf extracts and derivatives (tulsi) as distinct precursors. This is to see if the particles made just from the precursor quercetin have the same characteristics as the particles made from tulsi leaf extraction. AgNPs produced using the leaf extract and plain quercetin showed the same optical, morphological, and antibacterial characteristics, showing that the biomolecules (quercetin) contained in tulsi were largely responsible for reducing metal ions to MNPs [89]. Silver-mediated nanoparticles have been demonstrated to have a higher cytotoxicity in plants than gold, regardless of cell type. The size and shape of MNPs produced by plants had an impact on their cytotoxicity. Although cancer indications are acceptable, the therapeutic index of most nanoparticles is limited. MNPs synthesized from *Butea monosperma, Abutilon inducum, Indoneesiella echioides, Melia azedarach,* and *Gossypium hirsutum* are among the potential anticancer medications having an appropriate therapeutic indicator as a safety marker [90].

### 4.1. Applications in Dentistry

The antimicrobial characteristics of silver nanoparticles (AgNPs) have been thoroughly explored, and they can be used in a variety of dental procedures. *In vitro* studies demonstrate that AgNPs have a strong antibacterial effect when coupled with dental materials, including acrylic resins, nanocomposites, adhesives, resin comonomers, intracanal medications, and implant coatings. Furthermore, due to their anticancer capabilities, AgNPs are promising tools in managing oral malignancies [91]. In endodontics, AgNP was utilized as a disinfectant to eliminate bacteria, toxins, and debris from the root canal system to hinder microbial development and prevent infection. This fast expansion of the AgNP usage in endodontic substances has been mainly owing to its demonstrated antibacterial efficacy in about 650 bacterial species. Information that silver is less harmful to cells and tissues in nanoparticles further encouraged the usage of AgNPs to medicines and therapeutic uses. A study was performed to assess the synergistic effects of NPs and *Aloe vera* in root canal disinfection. In endodontic infections, *A. vera*-encapsulated nanomaterials showed durable antibacterial action [42]. Alveolar bone loss, a common condition, makes dental implant placement difficult. A barrier between the bone replacement and the gingiva that prevents fibrotissue ingrowth and bacterial infection and encourages bone development is crucial to alveolar ridge repair. Chen et al. demonstrated how AgNP-coated collagen membranes can help prevent infection after the insertion of bone grafts in alveolar ridge restoration [92].

Multiple studies have suggested their usage in a variety of formulations, with promising results in the treatment of *S. mutans*, with antibacterial activity 25 times stronger than chlorhexidine, as well as antiviral and antifungal activities [93]. Composite resin is now the most extensively utilized dental material to treat caries lesions, owing to its cosmetic and load-bearing qualities. Microleakage has been seen on composite repair edges, and oral microorganisms can colonize these perforations, resulting in secondary caries. Antimicrobial restorative materials have been created to prevent or reduce biofilm deposition, particularly by integrating AgNPs into composite resins and adhesive systems [94]. Dentures, which are usually made of polymethyl methacrylate (PMMA) acrylic resin, have a rough internal surface, along with other factors (such as poor hygiene and HIV infection), leading to the development of denture stomatitis. *Candida* species colonize denture surfaces, generating a biofilm that can induce the development of denture stomatitis [95]. Acosta-Torres et al. [63] created PMMA with 1 *μ*g/mL AgNPs and compared it to PMMA that had not been changed. PMMA AgNPs (*P* 0.05) specimens revealed reduced C. albicans adhesion than PMMA. Root canal fillings have been made from various materials, with gutta-percha being one of the most popular. Endodontic materials should ideally have some antibacterial action as bacterial removal in root canals is critical to treatment effectiveness. In an attempt to improve the characteristics of gutta-antibacterial percha, Iranian researchers created nanosilver gutta-percha. Gutta-percha coated with AgNPs is efficient against *E. faecalis, S. aureus*, *Candida albicans*, and *E. coli* [96]. Samiei et al. modified mineral trioxide aggregate (MTA) by adding AgNPs at 1% weight to boost its antibacterial activity. It was tested for its ability to eliminate oral bacteria and fungus. Compared to unmodified MTA, AgNP-containing MTA had a stronger antibacterial activity against *E. faecalis*, *Candida albicans*, and *Pseudomonas aeruginosa* [97]. Zhao et al. used silver nitrate immersion and UV radiation to insert AgNPs into titania nanotubes (TiO_2_-NTs) on Ti implants. The antibacterial action against *S. aureus* was tested, and the findings showed that planktonic germs were inhibited over the first days. Furthermore, Ti implants coated with AgNPs have shown to be able to inhibit bacteria adherence for up to 30 days, which is regarded enough time to avoid postinfection in the early stages [98].

Rodrigues et al. tested the antibacterial effectiveness including AgNP in an aqueous vehicle, chlorhexidine, and sodium hypochlorite against *Enterococcus faecalis* biofilm and infected dentinal tubules. The AgNP solution killed fewer bacteria than NaOCl but could dissolve more biofilm than chlorhexidine. AgNP irrigant was not as effective AgNP against *E. faecalis* as root canal therapy solutions [99]. Biomaterials containing AgNPs have been developed to prevent or minimize the production of biofilms. They have a unique antibacterial effect without altering the material's mechanical qualities due to their higher surface-to-volume ratio and tiny particle size. AgNPs have a unique feature that allows them to be used as fillers in various biomaterials, where they play an essential role in enhancing the characteristics [100]. These nanoparticles were employed in a two-way dental restorative treatment simultaneously. The use of glass ionomer cement in dentistry has found a significant restriction on poor wear and secondary caries caused by the buildup of bacterial colonies around the restoration when used at an early or aging stage. The produced silver nanoparticles were cemented with glass ionomer cement to meet the two limits. The addition of AgNPs to GIC improves the hardness of traditional GIC and, in turn, eliminates the limiting of secondary caries caused by bacterial colonies around the GIC-fixed restoration in postmedication [44]. Dental equipment and bandages have been made with AgNPs. The addition of AgNPs to orthodontic glue can improve or maintain the glue's shear bond strength while increasing its bacterial resistance [101]. Magalhães et al. found that including AgNPs into dental composites reduced microbial colonization of coating materials, enhancing antifungal capability [102]. Moreover, endodontic fillings containing AgNPs had an antimicrobial action that lasted for a long time against *Streptococcus milleri, Staphylococcus aureus*, and *Enterococcus faecalis* [103].

## 5. Gold Nanoparticles

A great deal of research and manufacturing approaches have been used to create gold nanoparticles (AuNPs) by various physical and chemical processes. Due to their unique physicochemical features and a wide variety of uses, numerous publications have been published in recent years on the synthesis and characterization of AuNPs. Physical methods of preparing metallic nanogold (e.g., laser ablation) produce GNPs with a narrow particle size distribution, but they are expensive and yield low. AuNPs can be made chemically (for example, sodium borohydride). Organic solvents' hazardous side effects and the toxic effects of reducing reagents employed in the chemical production of GNPs drew focus to the development of alternative green approaches [104]. AuNPs are a form of nanomaterial that can be readily produced using a one-step environmentally friendly green chemistry process. They are widely known for their biocompatibility and nontoxicity. AuNPs are an excellent contender for biological applications because of their characteristics [17].

Hyperaccumulators are plants that can absorb and collect metals from the water and soil.. Alfalfa may gather gold and store it as discrete pure metal nanoparticles in their leaves and stems' biomasses. Various plants, such as broth extracts of neem, *Aloe vera*, *Arena sativa*, alfalfa, wheat, geranium, *Hibiscus sabdariffa*, and lemongrass, have been effectively employed and reported for effective and quick extracellular synthesis of gold, silver, and copper nanoparticles in recent years. It possesses different actions that are ideal for therapeutic usage and broad applications in nanobiotechnology, and it possesses unique nanoscale gold properties [39]. Previous studies on AuNPs have included immune response augmentation, microbe detection, control, cancer cell photothermolysis, clinical chemistry, optical imaging of cancer cells using resonance scattering, targeted drug delivery, two-photon luminescence, and optical coherence tomography. Although AuNPs have the strongest antibacterial activity of all metal NPs, antibiotic-coupled AuNPs have shown the potential for photothermal protozoa and bacteria death. It was demonstrated that AuNPs conjugated with the anticancer drug 5-fluorouracil were more effective against bacteria and fungus than 5-fluorouracil alone. As a result, conjugated NPs can deliver antibiotics to a specific site [6]. AuNPs are suitable for biological applications due to their unique optical features derived from the SPR (surface plasmon resonance) phenomenon and their biocompatibility. AuNPs are shown to have a strong potential for photothermal cancer cell treatment. When AuNPs are subjected to electromagnetic radiation, the resonance of surface-conductive electrons absorbs the radiation in the visible and near-infrared ranges. Cancer cells are thermally degraded via the heat generated.

### 5.1. Applications in Dentistry

AuNPs have been used to treat gum disorders, dental cavities, tissue engineering, dental implantology, and cancer detection because of their nanostructures, huge surface volume, and biocompatibility. Because AuNPs have antifungal and antibacterial capabilities, they are employed to boost the effect in various biomaterials. They also improve material mechanical properties, resulting in improved results. To illustrate their therapeutic effects, they come in various sizes and concentrations. Because of their properties, AuNPs are a viable candidate for fillers in biomaterials [105].

#### 5.1.1. Dental Caries

AuNPs have a greater surface area because of their nanoscale, allowing for greater inorganic and organic chemical reactions. As a result, AuNPs can be used as a potential anticaries agent. It was discovered that including AuNPs into cavity disinfectants can improve the material's antibacterial activity and, as a result, reduce the risk of secondary caries when compared to conventional treatments [106].

#### 5.1.2. Dental Implants

AuNPs can be used as osteogenic agents for bone regeneration because of their biocompatibility and surface specificity. The osteoinductivity of *Salacia chinensis*-mediated AuNPs was investigated for usage as a green source osteoinductive biomaterial in implant dentistry by Jadhav et al. Plant-mediated AuNPs produced utilizing green chemistry have been shown to be biocompatible, environmentally friendly nanomolecules that stimulate bone formation and decrease bone resorption and may be utilized as an active bone inductive material during implant placement [55].

#### 5.1.3. Periodontal Disease

Periodontal disease diagnosis is critical for preventing further progression and providing proper treatment. AuNPs play a significant role in diagnosing periodontal disease due to their unique critical optical properties. According to the findings, the size and concentration of AuNPs have a favorable effect on the proliferation of these cells. As a result, AuNPs can be used as a source in tissue engineering to help mend diseased tissues [105].

#### 5.1.4. Stem Cell Technology

Because of their resemblance to a nanostructured environment, nanomaterials have piqued the interest of many tissue engineering experts. These nanomaterials have the ability to infiltrate the nuclei of cells and alter their functions. The effects of AuNPs on stem cells in tissue engineering have been investigated [31]. For the first time, Xia et al. tested the osteogenic induction potential of a new calcium phosphate cement containing AuNP-CPC on human dental pulp stem cells (hDPSCs). AuNPs increased hDPSCs' behavior on CPC, such as cell adhesion and proliferation, as well as osteogenic differentiation (approximately a 2- to 3-fold increase after 14 days) [107]. Wang concluded that using an eco-friendly, cost-effective, and accessible green synthetic technique, stable, biocompatible, and functional AuNPs may be effectively manufactured. The stability of AuNP colloid solution *in vitro* was shown to be outstanding in a range of blood components. They discovered that they may be utilized as a pain reliever and an osteoinductive adjuvant in the treatment of dental tissue implantation [54].

#### 5.1.5. Dental Materials

Dadkan et al [108] studied the effect of gold nanofiller particles on microtensile bond strength to dentin in an experimental bonding agent, as well as the optimal filler quantity required to achieve the maximum bond strength. The inclusion of AuNPs increases the flexural and tensile strength of the dental adhesive, with the optimal AuNP concentration resulting in the best mechanical properties. Its flexural and tensile strength optimum concentrations were 10*X* and 5*X*, respectively. AuNPs can function as a barrier to fracture development in terms of flexural strength, with higher concentrations having a stronger positive impact. At higher concentrations, NPs clump together, which might serve as a good starting point for a fracture [105].

#### 5.1.6. Diagnostic Imaging

Optical imaging is one of the most critical tools in biological research. Bio-optical imaging still has issues with resolution, sensitivity, speed, and penetration depth despite significant advancements. Because of their unique optical features, such as surface plasmon resonance, gold nanoparticles (AuNPs) can be easily employed to improve optical imaging through absorption, scattering, fluorescence, Raman scattering, and other means. According to the literature, AuNP-assisted bioimaging is a promising method for probing fundamental biological questions and detecting disorders early [109].

## 6. Iron Nanoparticles

Because of their exceptional physicochemical features, high magnetism, microwave absorption capabilities, low toxicity, and high catalytic activity, iron nanoparticles (INPs) are among the most intriguing new materials. Iron oxide nanoparticles (IONPs) (including magnetite: Fe_3_O_4_, hematite: −Fe_2_O_3_, and maghemite: −Fe_2_O_3_), iron oxide-hydroxide (FeOOH) nanoparticles, and zero-valent iron (ZVI) nanoparticles are the three principal categories of INPs [110]. Applications for these particles include delivery of the drug, magnetic targeting, heat exhaustion, heat ablation, stem cell trial and manipulation, gene editing, negative MRI contrast improvement, ferrofluids, preservation of food, bioprocess intensification, antimicrobial agents, environmental remedy, pigments, and lithium-ion batteries [111]. The particles are synthesized using a variety of physical and chemical processes, including the coprecipitation method, sol-gel method, microemulsion method, hydrothermal method, and solvothermal method. The green production of iron nanoparticles is gaining popularity as an environmentally friendly and cost-effective therapeutic method. Afsheen et al. used mango leaves, rose leaves, neem leaves, carom seeds, and clove buds to make iron nanoparticles in a zero-valent oxidation state by an eco-friendly green synthesis at 70°C temperature with steady stirring and atmospheric pressure. In combination with a specific proportion of polyvinylpyrrolidone (PVP) instead of polyvinyl alcohol (PVA), different plant extracts play an essential role in the reduction and stabilization of nanoparticles. The significant amount of PVP utilized prevented the particles from agglomerating and oxidizing. The presence of PVP allows particles at the micro-/nanoscale to maintain their crystalline structure after 3 to 4 months of preparation [71]. The primary mechanism of INP generation by plant extracts, including nucleation and particle development, is yet to be found. Phytochemicals (primary and secondary metabolites) in the plant extract, on the contrary, play a critical role in the biosynthesis of INPs, according to research. In fact, phenolic chemicals (polyphenols, flavonoids, tannic acid, and terpenoids) act as natural antioxidants that significantly decrease iron ions to INPs [112]. *Artocarpus heterophyllus* (jackfruit) peel extract was used to make iron nanoparticles (FeNPs). The peel's strong antioxidant content makes it a potential source of valuable biomolecules that can be used as bioreductants, capping agents, and stabilizing agents in green nanoparticle manufacturing. Apart from employing nontoxic reactant ingredients and being cost-effective, the approach makes use of trash and thereby lowers waste accumulation [113]. Green procedures are the most commonly utilized for the synthesis of INP utilizing the plant-mediated extract of *Citrus sinensis* since they are both environmentally benign and economically effective. Gram-negative bacteria and Gram-positive bacteria were used to investigate the antibacterial impact of biologically generated IONPs. These findings demonstrated that IONPs have a strong antibacterial potential as they inhibited bacterial strains in a substantial zone [112].

### 6.1. Applications in Dentistry

**Figure 7:**
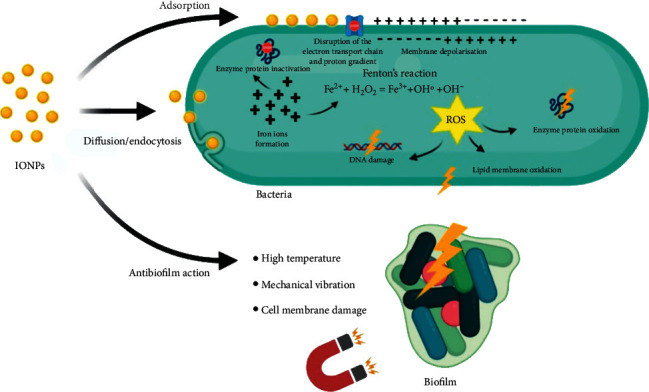
Antibacterial and antibiofilm mechanisms of iron oxide nanoparticles [25].

Iron oxide nanoparticles (IONPs), such as magnetite and maghemite magnetic nanoparticles, have received a lot of attention in recent years in various sectors. IONPs have been used in biomedicine for a wide range of applications, including diagnosis and treatment. These magnetic nanoparticles can be utilized as contrast in imaging. IONPs can also be employed as nanocarriers for delivering therapeutic drugs to desired cells *in vivo* due to their magnetic characteristics, low cost, and excellent biocompatibility [114]. Gao et al. described a new technique for controlling biofilms (plaques) based on catalytic nanoparticles (CAT-NPs) comprising biocompatible Fe_3_O_4_ with peroxidase-like activity that promotes extracellular matrix disintegration and bacterial mortality within acidic niches of caries-causing biofilms (Figure 7). They showed that CAT-NP combined with H_2_O_2_ successfully suppresses the onset and severity of dental caries *in vivo* while protecting normal tissues, using 1-minute topical daily treatments similar to those used in clinical settings [115]. The antibacterial activity of both natural and synthetic medications has been discovered to be improved by the synergy of INP. The action of common natural items against *S. mutans* was examined, including clove buds, neem leaves, and green tea leaves. To compare the effects of various combinations of treatments on *S. mutans*, researchers used the plant extract alone, a plant extract with INP, and the plant extract with INP and amoxicillin. When an antimicrobial agent is coupled with INP, the antimicrobial agent's action is enhanced [74].

## 7. Zinc Oxide Nanoparticles

Zinc is a trace mineral abundantly spread across the body tissue, which contributes to the catalytic activity of several enzymes [116]. Zinc oxide (ZnO) is a biocompatible semiconductor material that is utilized for different purposes to make various dental products such as zinc oxide eugenol, amalgam, ceramics, and dental cements. The Food and Drug Administration considers it to be one of the safest materials in the pharmaceutical industry. The intrinsic features of nanosized ZnO, such as its wide bandgap, high-exciton binding energy, high electronic conductivity, nontoxicity, and chemical durability, have piqued industrial interest. ZnONPs have unique optical features that make them suitable for use as a drug delivery system and anticancer, antibacterial, antidiabetic, and theragnostic tool [117]. Plants are the most popular source of NP synthesis because they allow for large-scale production. Phytochemicals such as polysaccharides, vitamins, amino acids, alkaloids, and terpenoids released by plants are used to reduce metal ions or metal oxides [118]. Plant extracts, such as phenols and flavones, can be used to make ZnONPs. The reducing and capping agents in the extracts, such as phenols and flavones, can stabilize the NPs by electrostatic, steric, hydration, and van der Waals forces. Plant extract-assisted biosynthesis is a reasonably straightforward process that can be completed in three steps. The preparation of the plant extract is usually the initial step. Then, as a precursor, zinc salts are added to plant extracts. Metal ions are reduced into NPs at this stage and then stabilized with reducing and capping agents. After various synthesis procedures such as high-temperature annealing, ZnONPs are created in the last stage [119]. Plants in the Lamiaceae family, including *Anisochilus carnosus, Plectranthus amboinicus,* and *Vitex negundo*, have been researched extensively for the NP formation of various sizes and shapes, including spherical, quasi-spherical, hexagonal, rod-shaped, and agglomerates. The size of produced NPs reduces when the content of a plant extract increases, according to the findings [120–122]. The size of CuNP produced from peel was larger, as validated by SEM and TEM studies, but the forms were similar (hexagonal and spherical). Agglomeration was seen in NPs made from extracts of *Moringa oleifera, Agathosma betulina, Pongamia pinnata, Plectranthus amboinicus, Nephelium lappaceum*, and *Calotropis gigantea* [123].

### 7.1. Applications in Dentistry

**Figure 8:**
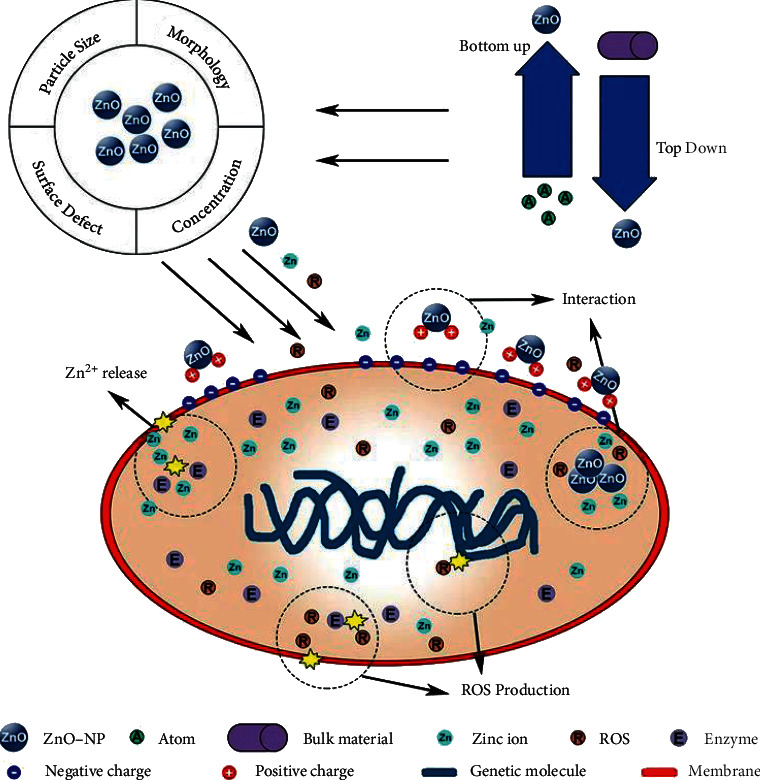
Antibacterial mechanism of ZnNPs [125].

Zn^2+^ ions disrupt bacterial enzyme systems by displacing magnesium ions required for dental plaque enzymatic activity. Tavassoli Hojati et al. [21] demonstrated that including ZnONPs into resin composites may considerably inhibit *S. mutans* strains without losing the resin's mechanical qualities. Another explanation could be due to electrostatic forces induced by light exposure, resulting in interactions between the nanoparticles and bacteria [124] (Figure 8). On *Streptococcus mutans*, the antibacterial effect of composite resin containing ZnONPs was much higher than that of composite resin containing AgNPs [126]. Implant failure is frequently caused by infections linked with the prosthesis and aseptic loosening. Improved antibacterial properties and osseointegration of orthopedic implants are critical. Zinc oxide nanoparticles (ZnONPs) are a form of zinc-containing metal oxide nanoparticle that has been extensively explored in a variety of sectors, including food packaging, pollution control, and biomedicine. Low toxicity and good biological functions, as well as antibacterial, anticancer, and osteogenic properties, characterize ZnONPs. Furthermore, ZnONPs may be made readily using a variety of ways. Green preparation approaches, for example, can improve the bioactivity of ZnONPs and increase their biological application potential [119]. Implants with ZnONP modifications have good antibacterial characteristics. Elizabeth et al. covered titania nanotubes and titania nanoleaves with ZnONPs. The antibacterial capabilities of modified samples were dramatically improved compared to pure nanopatterned materials [127]. By electrospinning polyetherimide (PEI) with various concentrations of ZnONPs, Artifon et al. were able to create a variety of ZnO/PEI scaffolds. The antibacterial action was more vital as the ZnONP level rose. ZnNP-modified implants can modulate the immune system and increase antibacterial characteristics, in addition to direct toxicity against microorganisms [128].

## 8. Titanium Oxide Nanoparticles

The entire biosynthetic process begins with mixing a simple precursor salt with the biological extract; the extract's metabolites can then reduce and stabilize the bulk metal into an elemental form through a series of mechanical stages. This biosynthetic approach has several advantages over chemical and physical procedures, and it has emerged as a simple, safe, and viable alternative. Aside from this, biological approaches can efficiently catalyze the synthesis process at any size and under any condition. Furthermore, NPs of controlled size and form can be made. Because of these advantages, several researchers have planned to investigate various species for their ability to manufacture TiO_2_ NPs [129]. Forming a solution of a titanium precursor with the required solvent is the most common way to make TiO_2_ nanoparticles. Ethanol and distilled water are the most commonly utilized solvents for this purpose. TTIP (titanium tetraisopropoxide), TiCl_4_ (titanium tetrachloride), and TiO(OH)_2_ are common titanium precursors used to greenly produce TiO_2_ nanoparticles (metatitanic acid or titanyl hydroxide). The synthesis procedures also use TiOSO_4_ (titanium oxysulfate) and TiO_2_ bulk particles; in fact, this is one of the advantages of green nanotechnology: water-soluble precursors can be used equally [130]. Plant components such as phenolic acids, alkaloids, proteins, enzymes, and carbohydrates govern the synthesis of NPs through reduction and stabilization mechanisms. To produce diverse forms of TiO_2_ NPs, a variety of plant species have been employed. When a precursor TiO_2_ salt is tainted with the plant extract, the reaction mixes heat up quickly. Then, the solution is constantly stirred at a moderate temperature. The initial synthesis indicator is a color change, which can be validated later using spectroscopic techniques. Several color indicators have been recorded in the production of TiO_2_ NPs, ranging from pale green to dark green [77]. The nanoparticles are then filtered, rinsed with distilled water, dried, and calcined. To eliminate organic groups, calcination is usually carried out at temperatures ranging from 400 to 800°C [130]. Ahmad et al. studied the antibacterial and antifungal properties of green-produced titanium dioxide (TiO_2_) nanoparticles utilizing *Mentha arvensis* leaf extract as the reducing agent and titanium tetraisopropoxide as the precursor. Green TiO_2_ nanoparticles showed promising antibacterial and antifungal action against various microorganisms [131]. TiO_2_ nanoparticles (NPs) were green-synthesized using the extracts of *Azadirachta indica* twigs*, Ficus benghalensis*, *Syzygium aromaticum*, *Mentha arvensis, Citrus aurantifolia, Echinacea purpurea*, and *Acanthophyllum laxiusculum* [75–77, 131].

### 8.1. Applications in Dentistry

Titanium dioxide nanoparticles (TiO_2_ NPs) are valuable additions in adhesives and composites because of their photocatalytic, antimicrobial, and UV-absorbing capabilities. However, the ROS created by photoactivated TiO_2_ NPs has been linked to toxicological concerns. Sun et al. revealed that acid-functionalized TiO_2_ NPs might be incorporated into dental resins that can be used as dental adhesives on human teeth. The ROS produced by these NPs when exposed to visible light can be used to boost the degree of vinyl conversion in resins, resulting in adhesives with improved shear bond strength to human teeth. The genotoxicity of the NPs and their potential for release from dental composites were investigated, and the results showed that there was a low danger of genotoxic effects [132]. When the mechanical properties of the composites were evaluated, it was discovered that using TiO_2_ as a reinforcing agent strengthened the polymer. The morphological observation revealed substantial adhesion between TiO_2_ and the polymer matrices, as well as a uniform distribution of TiO_2_ within the polymer matrix. The mechanical properties of TiO_2_ were improved by adequate compatibilization with the polymer matrix [133]. Titanium dioxide is an inorganic chemical that has recently received a lot of attention due to its photoactivity. TiO_2_ produces a variety of ROS after being exposed to UV radiation in aqueous solutions. The capacity to produce ROS and consequently induce cell death has been used in photodynamic therapy (PDT) to treat a variety of ailments ranging from psoriasis to cancer. TiO_2_ NPs have been investigated as photosensitizing agents for treating malignant tumors and photodynamic inactivation of antibiotic-resistant microorganisms. In PDT, TiO_2_ NPs can be employed as photosensitizers on their own, as well as in composites and mixtures with other chemicals or biomolecules. Furthermore, different chemical molecules can be grafted onto TiO_2_ nanoparticles to create hybrid materials. These nanostructures can show higher light absorption, allowing them to be used in medicine for focused therapy. Many titanium dioxide-based techniques were tested to improve the efficacy of anticancer and antibacterial medicines [134]. The extracts of *Azadirachta indica* twigs*, Ficus benghalensis,* and *Syzygium aromaticum* were used to make TiO_2_ nanoparticles (NPs). G-TiO_2_ NPs were investigated for antibacterial and antibiofilm properties against bacteria (*Streptococcus mutans* and *Citrobacter freundii*) and fungus (*Candida albicans*). According to this study, greenly produced TiO_2_ NPs have outstanding antibacterial and antibiofilm characteristics [75].

## 9. Copper Nanoparticles

**Figure 9:**
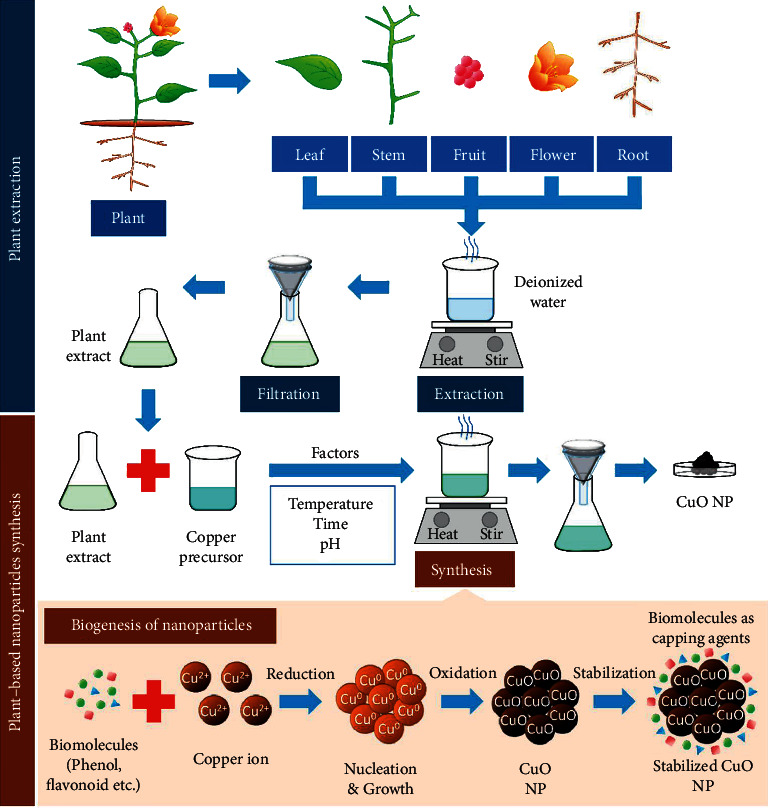
Plant-based green synthesis of copper oxide nanoparticles [136].

Copper nanoparticles (CuNPs) have garnered attention in the recent two decades because of their simple nature and the property of demonstrating a range of potentially beneficial physical properties depending on their size, shape, and composition. Natural plants have free radical scavengers that help prevent diseases including heart disease, cancer, arthritis, and liver disease and effectively reduce oxidative damage. Vital water in copper containers cleans water by killing bacteria species and strains and effectively eliminates bacteria, making it bactericidal. Furthermore, compared to other antibacterial agents such as gold and silver, copper is a less expensive option. Compared to other organic antibacterial agents, it exhibits antioxidant capabilities and longer shelf life. They have these unique physical, chemical, and biological features because of their highly distinctive crystal shape and high surface area-volume ratio [62]. Copper nanoparticles were created using both physical and chemical methods. The microemulsion technique is the most prevalent chemical strategy, although it is expensive and requires a high surfactant concentration. Physical methods for synthesizing nanoparticles include laser ablation, aerosol techniques, and radiolysis. However, these methods are less popular because of the high cost of instruments and high energy consumption. In the absence of a stabilizing agent, copper nanoparticles can be made via microwave irradiation. The inclusion of ascorbic acid during the synthesis of copper oxide results in the formation of nanoparticles. Because of their availability, cost-effectiveness, environmental friendliness, and lack of harmful byproducts, plants have been employed to synthesize metallic nanoparticles [135]. Mixing a known concentration of the plant extract with a known precursor concentration and heating the mixture to a defined temperature with continuous stirring at a set period is one of the most popular methods for generating Cu and CuO NPs (Figure 9) [136]. The medical qualities of the plant extract are seen to protect the NPs generated from it, which could be used in medicine, targeted drug delivery, and cosmetic applications. CuNPs have also gained interest due to their potential industrial applications, such as gas sensors, catalytic processes, high-temperature superconductors, and solar cells, as well as their applications in wound dressings and biocidal qualities. Antibiotics use CuNPs because of their remarkable physical characteristics. They are used as a bactericidal agent to coat medical equipment, such as heat transfer systems, antimicrobial materials, superstrong materials, sensors, and catalysts, due to their disinfecting characteristics and matrix stability. The bactericidal impact of NPs has been improved because of their tiny size [137]. CuNP has been synthesized using extracts of various plants such as *Celastrus paniculatus, Cardiospermum halicacabum, Zingiber officinale, Eryngium caucasicum, Plectranthus amboinicus, Azadirachta indica, Punica granatum, Eclipta prostrata, Citrus medica* Linn., and *Madhuca longifolia* [28, 60–62, 64–69].

### 9.1. Applications in Dentistry

Unlike silver, ions' release is not the most critical factor in copper nanoparticles' bactericidal activity. Other parameters, such as the nanoparticles' oxidation state, size, and crystalline structure, significantly impact the process. CuNPs' bactericidal action against *Aggregatibacter actinomycetemcomitans* (one of the principal pathogens responsible for producing localized aggressive periodontitis) and cytocompatibility make them a good candidate for use as an anti-peri-implantitis agent in oral implants [138]. Copper oxide nanoparticles made from *Aloe vera* gel have been found to have excellent anticariogenic properties and can be used in a variety of dental applications [36]. To prevent and minimize the occurrence of secondary caries, glass ionomer cements (GICs) with an excellent fluoride-ion releasing function have been used. The antibacterial characteristics of the copper-doped glass ionomer-based materials were dramatically increased, and collagen degradation was significantly reduced. The use of copper-doped glass ionomer-based materials for composite restore may assist them to live longer because of its increased antibacterial capacities and decayed collagen breakdown [139]. CuNP applied to adhesives at a concentration of up to 0.5 wt% can offer antibacterial properties and prevent adhesive interface degradation without affecting the formulation's mechanical capabilities, according to Gutiérrez et al. [140].

## 10. Future Prospects

Herbal medication, nanometer-sized, has a bright future in enhancing biological activity and overcoming the issues associated with chemical/synthetic pharmaceuticals. As a result, the use of green pharmaceuticals in nanodrug delivery systems improves the usage of herbal plants and aids in treating numerous ailments. Plant nanoparticles can be used to a greater extent to prevent oral diseases, dentures, and implants, oral cancer prevention and treatment, and oral health care. Furthermore, substantial research on the numerous chemicals found in herbal remedies, as well as their subsequent nanoparticle manufacturing, should be done. Herbal nanomedicine technologies must be thoroughly investigated for efficacy and application in dentistry. *In vivo* testing should be done on all plant nanoparticles that demonstrate anticancer potential in diverse malignancies. Plant nanoparticles with antioxidant properties should also be tested for a variety of dental uses.

## 11. Conclusion

Since ancient times, herbal medicine has been utilized to treat oral/dental diseases and provide everyday care. Plant-mediated biogenic metal nanoparticles can overcome the drawbacks of herbal treatments, making the development of herbal medicine-incorporated nanoparticle formulations in dentistry a novel breakthrough. The use of medicinal plant extracts in the green production of metal nanoparticles such as silver, copper, and gold nanoparticles has been shown to be effective in treating a variety of oral/dental diseases, even outperforming conventional materials. These are also found in toothpaste and mouthwash, as well as other dental care products. However, there is a lack of knowledge about the safety of nanomaterials, necessitating more research. Many concerns, including medication resistance, could be addressed using nanoparticles and a combination of plant extracts. Herbal medicines are a suitable option for allopathic drugs in dentistry since they lessen the adverse effects of chemicals and antibiotics. Therefore, herbal extracts are precious in dental care because of their safety, naturalness, and cost-effectiveness. In this instance, educating people about the advantages of utilizing herbal treatments to avoid oral diseases will be quite valuable. Nanotechnology is expected to be used in all dental applications because it effectively addresses herbal medicine's limitations such as low oral absorption, low water solubility, poor bioavailability, physical instability, and slow and toxic pharmacological actions.

## Figures and Tables

**Table 1:** Green synthesis of plant-mediated metal nanoparticles which have been used in dentistry.

NP	Plant	Objective	Outcome	NP size	Year/ref
AgNP	*Acacia senegal*	To make AgNPs, the unique technique of utilizing tree exudates containing a high quantity of polysaccharides in gum Arabic (GA) produced from the *Acacia senegal* (L) wild tree was utilized. The effect of synthesized AgNPs was evaluated against *S. mutans* isolates utilizing well diffusion and microdilution methods.	The synthesized NPs' strong antibiotic activity against *S. mutans* provides the door for creating new dental care products. The tiny size of the NPs further aids their application in COVID-19 pandemic containment.	<10 nm (spherical shape)	2021/[34]
AgNP	*Camellia sinensis* (green tea)	This work aimed to make light-colored Ag-SiO_2_ nanoparticles by synthesizing AgNPs from the green tea (GT) extract and coating their surfaces with silica.	Ag-SiO_2_ NPs were shown to have strong antibacterial activity against *S. mutans*, with a 600 g/mL MIC and biofilm inhibition of around 44% (*p* 0.05). Both NPs did not cause cytotoxicity at the MIC doses.	11 nm (spherical shape)	2020/[35]
AgNP	*Azadirachta indica* and *Aloe vera*	The purpose of this study was to see if AgNP made from neem and *Aloe vera* had any antibacterial activity against four dental pathogens.	AgNPs made from neem and *Aloe vera* have been found to be efficient against *Streptococcus mutans* and *Pseudomonas* species	_	2019/[36]
AgNP	*Viola serpens*	The objective of this study was to see if silver nanoparticles produced from the *Viola serpens* plant have antibacterial and antiplaque capabilities. Antibacterial tests were used to assess the efficacy of biologically generated AgNPs against recovered isolates.	Compared to the reference medicine, they were shown to be relatively effective against the three strains of *S. mutans*.	_	2018/[37]
AgNP	*Curcuma aromatica*	To produce AgNPs, researchers utilized *Bacillus amyloliquefaciens* SJ14 culture (MAgNPs) and extracted from *Curcuma aromatica* rhizome (CAgNPs). MIC, MBC, and antibiofilm properties of AgNPs against *S. mutans* were investigated. PMMA/MAgNPs and PMMA/CAgNPs nanocomposite thin films were tested for antimicrobial and antibiofilm properties.	When compared to CAgNPs, MAgNPs were found to have better antibacterial action. At doses of 3 g/mL and 50 g/mL, respectively, MAgNPs and CAgNPs inhibited *S. mutans* biofilm formation by 99 and 94 percent, respectively. The microbicidal activity of the PMMA/MAgNP thin film was found to be more significant.	10–30 (irregular shape)	2018/[38]
AgNP	*Oryza sativa* L. (rice)	Rice bran (RB), rice husk [39], and rice germ (RG) aqueous extracts were evaluated for their ability to function as reducing agents in the generation of AgNPs.	The findings imply that aqueous extracts of RB, RH, and RG might be utilized as reducing agents in AgNP production.	346.4 ± 36.8 nm	2018/[40]
AgNP	*Punica granatum* L. (pomegranate)	The objective was to develop, characterize, and test novel nanocomposites containing AgNPs that were either associated or not with calcium glycerophosphate for antibacterial and antibiofilm characteristics.	All of the extracts utilized were able to produce AgNPs. Antimicrobial and antibiofilm activities of composites produced with peel extracts were greatest against both bacteria tested, and they performed comparably to or better than chlorhexidine.	50 nm	2018/[7]
		Following treatments, the MBC/MFC and biofilm density were used to assess antimicrobial and antibiofilm capabilities against *Candida albicans* and *Streptococcus mutans.*			
Ag_2_O NP	*Ficus benghalensis*	The antibacterial activity of Ag_2_O NPs produced with the *Ficus benghalensis* prop root extract (FBPRE) as a RA and SA is described and investigated, as well as their antibacterial efficacy against dental bacterial strains.	The combination of FBPRE and Ag_2_O NPs has good antibacterial activity against *Streptococcus mutans* and *Lactobacilli* sp., two dental pathogens.	42.7 nm	2017/[41]
AgNP	*Aloe vera*	The purpose of this study was to compare the antibacterial effectiveness of *Aloe vera* nanoparticles to calcium hydroxide in the treatment of chronic endodontic infections.	The antibacterial activity of AgNPs from *A. vera* should prompt further study into their application as an intracanal medicament in root canal therapy.	_	2017/[42]
AgNP	*Tragia involucrata*	The AgNPs were made using a simple green technique using an aqueous extract of *T. involucrata*. UV spectroscopy, particle size measurement, zeta potential, and TEM were used to demonstrate the production of AgNPs. A single gel diffusion technique was used to test the extract's *in vitro* struvite growth inhibitory efficacy.	The findings suggested that an aqueous extract of *T. involucrata* and its AgNPs might be used to treat patients with recurring stones.	47 nm	2017/[43]
AgNP	*Mangifera indica*	A novel green synthesis method was utilized to make silver nanoparticles from leaves of *Mangifera indica*. The antibacterial activity of AgNPs is tested on *Escherichia coli* and *Staphylococcus aureus* germs, and the hardness of AgNP-reinforced GIC is compared to ordinary GIC and microsilver-reinforced GIC.	As seen by the results, the AgNP-reinforced GIC had a much higher hardness, and the AgNPs were shown to have an acceptable antibacterial activity.	32 nm	2017/[44]
AgNP	*Prunus japonica*	A simple room-temperature approach was utilized to produce AgNPs from AgNO_3_ at a low cost and in an ecologically acceptable way using the *Prunus japonica* leaves' extract as an RA.	The produced AgNPs have antibacterial efficacy against tested bacteria to varying degrees, with *Proteus vulgaris* having the highest activity.	24 nm spherical	2017/[45]
AgNP	*Ficus religiosa*	AgNPs were produced and described utilizing the *Ficus religiosa* leaf extract.	In several cancer cell lines, the AgNPs exhibited strong antibacterial action as well as cytotoxicity.	21 nm	2017/[15]
AgNP	*Ficus carica*	They suggested utilizing dried fig (*Ficus carica* L.) fruit extract as an RA and CA to make AGNPs from a 1 mM AgNO_3_ solution in a cost-effective and ecologically acceptable manner. Nanoparticles were studied using UV absorption spectroscopy and SEM.	Silver nanoparticles were successfully synthesized using the dried fruit extract of *Ficus carica*. According to the anticancer test, the AgNPs reduced by the fig extract showed a strong anticancer effect against MCF7 cells, and further animal acute toxicity experiments indicate that the aforementioned AgNPs are toxicologically harmless when given orally.	54–89 nm	2017/[46]
AgNP	*Psoralea corylifolia*	Biologically produced AgNPs from the *Psoralea corylifolia* seed extract using a 1 mM AgNO_3_ solution. The synthesized herbal-mediated AgNPs were submitted to different characterization procedures and evaluated in healthy female albino mice for *in vitro* antidiabetic efficacy and potentially harmful consequences.	This research might lead to the creation of valuable nanomedicines to treat a variety of disorders and highlight AgNPs' safety and biocompatibility within biological cells.	15–25 nm (average: 18 nm)	2017/[47]
AgNP	*Azadirachta indica*	The aim was to synthesize AgNPs using the *Azadirachta indica* aqueous leaf extract and investigate their antibacterial effects on human pathogenic *Escherichia coli* and *Staphylococcus aureus.*	Both Gram-positive and Gram-negative pathogens were susceptible to the AgNPs.	34 nm	2016/[48]
		Characterization of produced nanoparticles using various methods.			
AgNP	*Ficus benghalensis* and *Azadirachta indica*	The agar well diffusion technique was used to test the antibacterial activity of green-produced AgNPs against some bacteria. Cell viability was studied to assess the cytotoxic effect of green-produced AgNPs.	Gram-negative and Gram-positive microorganisms exhibited potential antibacterial action against the NPs. The produced AgNPs demonstrated antiproliferative efficacy against the MG-63 osteosarcoma cell line in a dose-dependent manner.	40–50 nm	2016/[49]
AgNP	*Salix alba*	DAD was used to test the antibacterial activity of these biologically produced silver nanoparticles.	The antibacterial efficacy of these manufactured silver nanoparticles was demonstrated against bacteria isolated from dental plaque.	29–35 nm	2016/ [50]
AgNP	*Emblica officinalis*	The AgNPs were produced using *E. officinalis* (fruit extract). The nanoparticles were analyzed using UV-Vis spectrophotometers, FTIR measurements, and SEM and XRD to determine the presence of *E. officinalis* biomolecules encapsulated in AgNPs.	The results showed that the *E. officinalis* fruit extract is an excellent bioreductant for the production of AgNPs. The produced AgNPs were found to inhibit and have considerable antibacterial activity against bacterial strains.	15 nm	2015/[51]
		DAD was used to evaluate the antibacterial activity of the produced AgNPs against the isolates.			
AgNP	*Justicia glauca*	The antimicrobial activities of green-synthesized AgNPs and drug-blended AgNPs against dental caries and periodontal disease-causing microorganisms were tested.	The antibacterial and antifungal activities of AgNPs and drug-mixed AgNPs were considerable. The MIC of AgNPs against these bacteria was found to be between 25 and 75 g/mL.	10–20 nm	2015/[52]
AgNP	*Eucalyptus oleosa*	Aqueous AgNO_3_ was mixed with the *E. oleosa* leaf extract under nonphotomediated conditions to produce colloidal silver nanoparticles.	This study revealed that silver nanoparticles may be produced by adjusting key parameters, and executing the synthesis method under ideal conditions resulted in silver nanoparticles with an average size of 21 nm.	21 nm	2015/[53]
AuNP	*Anogeissus latifolia*	This study aimed to assess the osteoinductive capacity and analgesic effects of gold nanoparticles (AuNPs) made with phytochemicals from *Anogeissus latifolia.*	This study found that green-synthesized AuNPs are effective analgesic and bone-inducing agents in implantation therapy.	50–60 nm (crystalline)	2020/[54]
AuNP	*Salacia chinensis*	They looked at the osteoinductive properties of gold nanoparticles mediated by *Salacia chinensis* in implant dentistry.	The findings revealed that AuNPs may be utilized as an efficient bone inductive agent during dental implant therapy since they are stable, biocompatible, and environmentally friendly.	1.5 ± 0.8 nm (distorted spherical shape)	2018/[55]
AuNP	*Indigofera tinctoria*	On the lung cancer cell line A549, the cytotoxic impact of the *I. tinctoria* leaf extract and nanoparticles was investigated. The agar well diffusion technique was used to assess antimicrobial activity against bacterial and fungal strains. Using the DPPH technique, the antioxidant activity of produced nanoparticles was determined.	It was discovered that when the quantity of nanoparticles increases, cell viability declines, and nanoparticles have a more damaging effect on cancer cells than the pure leaf extract. The nanoparticles produced had strong antibacterial activity against all investigated microbial strains to various degrees. It was discovered that nanoparticles have more antioxidant activity than the leaf extract.	AuNP: 6–29 nm (19.73 nm)	2018/[56]
AgNP				AgNP: 9–26 nm (16.46 nm)	
AuNP	*Alternanthera philoxeroides*	They used *A. philoxeroides* leaves to make phytochemically gold nanoparticles and tested the positive effects of these biologically synthesized nanoparticles against various microbial strains.	Overall, the findings point to a successful production of green nanoparticles as well as an enhancement in gold nanoparticle antibacterial efficacy.	72.11 ± 2.87 nm	2018/[57]
AuNP	*Justicia glauca*	The research focuses on *Justicia glauca* (aqueous leaf extract) mediated AuNP production at room temperature by treating chloroaurate ions, which has an antagonistic impact against oral pathogenic bacteria and fungi when taken with the medicines azithromycin and clarithromycin.	Against oral infections, AuNPs and drug-conjugated AuNPs demonstrated potential antibacterial and antifungal action. MIC values of biogenic AuNPs against various oral infections were found to be in the range of 6.25–25 g/mL.	32.5 ± 0.25 nm	2017/[6]
AuNP	*Panax ginseng*	The study tested the antibacterial uses of silver nanoparticles against pathogenic microbes and developed a simple technique for the green synthesis of silver and gold nanoparticles using the fresh root extract from a four-year-old *Panax ginseng* plant.	Techniques utilizing various equipment were used to characterize the biosynthesized AuNP and AgNP. Furthermore, silver nanoparticles have antibacterial properties.	AuNP: 10–40 nm (spherical)	2016/[58]
AgNP				AgNP: 10–30 nm (spherical)	
AuNP	*Stevia rebaudiana*	The possibility of utilizing the *Stevia rebaudiana* (SR) leaf extract to reduce gold ions to nanoparticles has been investigated. The aqueous extract for this investigation was made from *Stevia* leaves. Different approaches were used to characterize gold nanoparticles.	The findings show that the leaf extract of *S. rebaudiana* may produce gold nanoparticles (SR).	5–20 nm	2015/[59]
CuNP	*Celastrus paniculatus*	The antifungal activity of the *C. paniculatus* leaf extract against *Fusarium oxysporum* and its photocatalytic efficiency in the breakdown of organic dye were all evaluated in this work.	CuNPs were effectively synthesized via a green method and utilized as photocatalysts and antifungal agents, according to the findings.	2−10 nm (spherical)	2020/[60]
CuNP	*Cardiospermum halicacabum*	The present study synthesized CuNPs by a GS method with the *Cardiospermum halicacabum* leaf extract. The antibacterial and antibiofilm analyses were conducted to confirm their aptitude for biomedical applications.	CuNPs were shown to inhibit biofilm formation by adhering to the cell wall and disrupting their growth and development.	30–40 nm (hexagonal shape)	2020/[61]
CuNP	*Zingiber officinale*	This research aimed to learn more about the antioxidant effects of copper nanoparticles made from dried ginger.	Copper nanoparticles made with *Zingiber* have strong free radical scavenging activity, making them antioxidants with various medicinal and dental uses.	Not measured	2020/[62]
CuNP	*Eryngium caucasicum*	CuNPs were synthesized using an aqueous extract of *Eryngium caucasicum* Trautv., and different methods were used to validate the results [63].	According to the findings, the use of an aqueous extract of *E. caucasicum* Trautv. offered considerable promise for establishing a clean, simple, cost-effective, and efficient technique for green copper nanoparticle production.	<40 nm (nearly spherical)	2020/[64]
CuNP	*Plectranthus amboinicus*	They performed an environmentally friendly copper nanoparticle synthesis technique utilizing the *Plectranthus amboinicus* leaf extract, a simple and ostentatiously fast approach that yields stable nanoparticles.	This approach has been shown to be cost-effective, simple to use, and free of contaminants.	16–25 nm (crystalline)	2020/[65]
CuO NP	*Madhuca longifolia*	*Madhuca longifolia* plant extract, which works as a nontoxic reducing agent, has been used to establish an effective and environmentally acceptable approach to the green production of CuO nanoparticles (NPs).	CuO NPs exhibited strong antibacterial action against *E. coli*, *S. aureus*, and *B. subtilis* bacteria, with findings, compared to ampicillin and tetracycline.	30–120 nm	2019/[66]
CuNP	*Azadirachta indica*	CuNPs were produced using *Azadirachta indica* leaf broth, and the influence of various reaction parameters on the conversion rate and shape of the CuNPs was investigated.	The following were the optimal conditions for synthesis: 20% leaf broth, [CuCl_2_]_ _= 7.5 103 M, pH 6.6, and temperature 85°C. The current research might have a huge influence on the ability to produce metallic nanoparticles on a large scale in the near future.	48 nm (crystalline, cubical shape)	2018/[67]
CuNP	*Punica granatum*	The goal of this study was to use plant extracts as RA and SA in the GS of CuNPs. It would also look at the antibacterial properties of the CuNPs that had been produced.	The copper nanoparticles can be easily produced using the *Punica granatum* fruit rind extract and may be employed as effective antibacterial agents against *Staphylococcus aureus.*	56–59 nm	2018/[68]
CuNP	*Eclipta prostrata*	This study described the invention of a technique for making CuNPs by combining copper acetate solution with the *Eclipta prostrata* leaf extract without the need for a surfactant or external energy.	The antioxidant potential of the biosynthesized CuNPs was impressive. Similarly, *in vitro* anticancer experiments revealed that produced CuNPs had cytotoxicity against HepG2 cells. The results of this work show that biosynthesized CuNPs made from *E. prostrata* extracts might be utilized for medicinal purposes, making them a potential nanomaterial.	23–57 nm (face-centered cubic structure)	2017/[28]
CuNP	*Citrus medica* Linn.	They developed a safe and low-cost technique for the production of CuNPs utilizing citron juice. CuNPs were tested for antibacterial activity.	CuNPs produced by this method showed considerable inhibitory efficacy against tested microorganisms.	20 nm	2015/[69]
Fe_3_O_4_ NPs	*Euphorbia hirta*	In the green production of magnetic iron oxide nanoparticles, they employed the *Euphorbia hirta* leaf extract (Fe_3_O_4_ NPs)	The antibacterial activity of the produced iron oxide nanoparticles was tested against various bacterial and fungal pathogens, with highly encouraging results.	25–80 nm (cavity-like structure)	2020/[70]
FeNP	Rose, *Azadirachta indica* (neem), carom, *Syzygium aromaticum* (clove)	These particles were made using mango leaves, rose leaves, neem leaves, carom seeds, and clove buds in an environmentally friendly green synthesis process at 70°C with continual stirring and atmospheric pressure.	The size of Fe particles grew larger as the concentration of polyvinylpyrrolidone (PVP) rose, according to the findings of the experiments. The presence of PVP allows particles at the micro-/nanoscale to maintain their crystalline structure after 3 to 4 months of preparation.	12–28 nm (crystalline)	2019/[71]
FeONP	*Moringa oleifera*	They used the *Moringa oleifera* leaf extract to make FeONPs and examined their fluoride ion adsorption potential, comparing their effectiveness to that of a commercially available adsorbent.	The regeneration process revealed that FeONPs may be reused three times in the fluoride ion adsorption method. Due to its adsorption characteristics and the shortest contact time to reach equilibrium, FeONP is a potential material for fluoride ion removal.	4.14 nm	2018/[72]
Fe_3_O_4_ NPs	*Couroupita guianensis*	They have proposed utilizing the *C. guianensis* aqueous fruit extract to produce magnetic Fe_3_O_4_ NPs for antibacterial and theranostic cancer applications.	These Fe_3_O_4_ NPs demonstrated outstanding bactericidal activity against various human diseases, demonstrating their antibacterial potential. Fe_3_O_4_ NPs had a substantial dose-dependent cytotoxic effect on treated human hepatocellular carcinoma cells (HepG2).	17 ± 10 nm (crystalline)	2017/[73]
FeNP	*Syzygium aromaticum* (clove), *Azadirachta indica* (neem), *Camellia sinensis* (green tea)	The activity of popular natural items such as clove buds, neem leaves, and green tea leaves against *S. mutans* was investigated. *S. mutans* was treated with several combinations of therapies.	The study found that adding FeNP to an antibacterial treatment boosts its action.	_	2017/[74]
TiO_2_ NP	*Azadirachta indica* twigs*, Ficus benghalensis*, and *Syzygium aromaticum*	TiO_2_ nanoparticles (NPs) were green-synthesized utilizing *Azadirachta indica* twigs, *Ficus benghalensis,* and *Syzygium aromaticum* extracts.	According to this study, greenly produced TiO_2_ NPs have outstanding antibacterial and antibiofilm characteristics.	18.95 nm (crystalline)	2020/[75]
		TiO_2_ NPs were evaluated for antibacterial and antibiofilm properties against bacteria (*Streptococcus mutans* and *Citrobacter freundii*) and fungi (*Candida albicans*)			
TiO_2_ NP	*Citrus aurantifolia*	This article focused on the production of TiO_2_ fillers and their potential use in light-curing dental composite materials.	The findings revealed that TiO_2_ nanohybrids might be utilized as excellent fillers for light-curing dental nanohybrid composite materials to improve their physical characteristics, in addition to their antibacterial, hydrophilic, and self-cleaning capabilities.	30–40 nm (crystalline)	2019/[76]
TiO_2_ NP	*Echinacea purpurea*	An aqueous solution of the *Echinacea purpurea* herbal extract was used as a bioreduction in this work to biosynthesize titanium dioxide nanoparticles.	UV-Vis spectroscopy, FTIR spectroscopy, and TXRF were used to establish the existence of TiO_2_ nanoparticles.	120 nm	2017/[77]
TiO_2_ NP	*Acanthophyllum laxiusculum*	The aqueous extract of *Acanthophyllum laxiusculum* was used in this work to create green or environmentally friendly TiO_2_ NPs. The sol-gel process, one of the most extensively utilized in the nanofield, was employed to make titanium dioxide nanoparticles.	UV-Vis absorption spectra were used to determine TiO_2_ nanospheres and then confirmed using diffuse reflectance spectroscopy.	25 nm (crystalline)	2016/[78]
ZnONP	*Deverra tortuosa*	Using the MTT test, the potential anticancer activity was examined *in vitro* against two cancer cell lines (human colon adenocarcinoma “Caco-2” and human lung adenocarcinoma “A549”), as well as human lung fibroblast cell line (WI38).	Against the two cancer cell lines tested, both aqueous extract and ZnONPs demonstrated significant selective cytotoxicity.	9–31 nm (15.41)	2020/[79]
ZnONP	*Dysphania ambrosioides*	ZnONPs were synthesized utilizing a green synthesis technique, including *Dysphania ambrosioides* extract.	This report discussed the structural properties of zinc oxide nanoparticles (ZnONPs)	5–30 nm (quasi-spherical)	2020/[80]
		Their antibacterial properties were tested against bacteria such as *S. aureus, S. epidermidis, E. coli,* and *Pseudomonas aeruginosa*, as well as bacteria commonly found in human mouths and linked to dental conditions.	According to the antibacterial test, most of the bacterial strains utilized in this investigation were susceptible to synthetic and commercial NPs, with *Prevotella intermedia* being the most sensitive to ZnONPs.		
ZnONP	*Sesamum indicum* L.	They described that entire vegetative portions of *Sesamum indicum* L. were used to produce ZnONPs. In this work, aqueous extracts of several sections of *S. indicum* were utilized to make nanoparticles.	This study will result in the development of cost-effective ZnONP synthesis with possible further exploration to serve humankind.	_	2019/[81]
ZnONP	*Juglans regia* L.	*Juglans regia* L. leaf extract was used in the green production of zinc oxide nanoparticles. The characteristics of zinc oxide nanoparticles produced using both green and chemical techniques were compared.	ZnO nanoparticles produced using a green approach had a more substantial antibacterial impact than chemically manufactured ZnO nanoparticles. The cytotoxicity of ZnO nanoparticles generated using the green approach was lower than that of chemically synthesized ZnO nanoparticles.	45–65 nm (spherical)	2019/[82]
ZnONP	*Salvadora persica*	Under ambient circumstances, *Salvadora persica* extract was used as a mediator in the production of ZnONPs. The MTT assay was applied to test the cytotoxic activity of the biosynthesized nanoparticles against the HT-29 cancer cell line.	UV-Vis investigations of these specific nanoparticles indicated the production of ZnO nanoparticles.	60–130 nm (hexagonal)	2019/[83]
			The findings revealed that the toxicity of manufactured nanoparticles is proportional to their concentration.		
ZnNP	*Lavandula vera*	The ZnNPs were produced utilizing a GS method in the presence of the *Lavandula vera* leaf extract. The median fatal dose of ZnNPs and subacute toxicity were established using a variety of tests.	The results showed that changes in OS were unrelated to the caspase pathway and that the dosage of biogenic ZnNPs with no observable adverse effects (NOAEL) in a 14-day subacute toxicity trial was less than 1 g/kg.	30–80 nm	2019/[84]
ZnONP	*Costus pictus* D. Don	Green-synthesized ZnONPs were tested against bacteria and two fungal species, including *Candida albicans*. The antitumor effect of the produced ZnONPs was investigated in mice with Dalton's lymphoma ascites (DLA).	When tested using the DAD technique, the green-synthesized ZnNPs demonstrate good antibacterial efficacy against bacterial and fungal species. The ZnONPs have been shown to have an anticancer effect against DLA cells.	20–80 nm (40 nm)	2018/[85]
